# Ten Novel Mutations in Chinese Patients with Megalencephalic Leukoencephalopathy with Subcortical Cysts and a Long-Term Follow-Up Research

**DOI:** 10.1371/journal.pone.0157258

**Published:** 2016-06-20

**Authors:** Binbin Cao, Huifang Yan, Mangmang Guo, Han Xie, Ye Wu, Qiang Gu, Jiangxi Xiao, Jing Shang, Yanling Yang, Hui Xiong, Zhengping Niu, Xiru Wu, Yuwu Jiang, Jingmin Wang

**Affiliations:** 1 Department of Pediatrics, Peking University First Hospital, Beijing 100034, China; 2 Department of Pediatrics, Beijing Tian Tan Hospital, Capital Medical University Beijing 100050, China; 3 Department of Radiology, Peking University First Hospital, Beijing 100034, China; 4 Department of Neurology, Shanxi Dayi Hospital, Taiyuan 030000, Shanxi Province, China; 5 Department of Neurology, First Hospital of Shanxi Medical University, Taiyuan 030001, Shanxi Province, China; Odense University Hospital, DENMARK

## Abstract

**Objective:**

Megalencephalic leukoencephalopathy with subcortical cysts (MLC, OMIM 604004) is a rare neurological deterioration disease. We aimed to clarify clinical and genetic features of Chinese MLC patients.

**Methods:**

Clinical information and peripheral venous blood of 20 patients and their families were collected, Sanger-sequencing and Multiple Ligation-dependent Probe Amplification were performed to make genetic analysis. Splicing-site mutation was confirmed with RT-PCR. UPD was detected by haplotype analysis. Follow-up study was performed through telephone for 27 patients.

**Results:**

Out of 20 patients, macrocephaly, classic MRI features, motor development delay and cognitive impairment were detected in 20(100%), 20(100%), 17(85%) and 4(20%) patients, respectively. 20(100%) were clinically diagnosed with MLC. 19(95%) were genetically diagnosed with 10 novel mutations in *MLC1*, *MLC1* and *GlialCAM* mutations were identified in 15 and 4 patients, respectively. Deletion mutation from exon4 to exon9 and a homozygous point mutation due to maternal UPD of chromosome22 in *MLC1* were found firstly. c.598-2A>C in *MLC1* leads to the skip of exon8. c.772-1G>C in *MLC1* accounting for 15.5%(9/58) alleles in Chinese patients might be a founder or a hot-spot mutation. Out of 27 patients in the follow-up study, head circumference was ranged from 56cm to 61cm in patients older than 5yeas old, with a median of 57cm. Motor development delay and cognitive impairment were detected in 22(81.5%) and 5(18.5%) patients, respectively. Motor and cognitive deterioration was found in 5 (18.5%) and 2 patients (7.4%), respectively. Improvements and MRI recovery were first found in Chinese patients. Rate of seizures (45.5%), transient motor retrogress (45.5%) and unconsciousness (13.6%) after head trauma was much higher than that after fever (18.2%, 9.1%, 0%, respectively).

**Significance:**

It’s a clinical and genetic analysis and a follow-up study for largest sample of Chinese MLC patients, identifying 10 novel mutations, expanding mutation spectrums and discovering clinical features of Chinese MLC patients.

## Introduction

Megalencephalic leukoencephalopathy with subcortical cysts (MLC, OMIM 604004) is a rare autosomal recessive or dominant heterogeneous neurodegenerative disease, which was firstly reported in 1995[1]. Classic phenotype is characterized by infant-onset macrocephaly, moderate or severe gross motor development delay, normal or mildly impaired mental development, progressive neurological deterioration, with ataxia, spasticity, seizures and sometimes extrapyramidal findings[1–2]. Magnetic resonance imaging (MRI) shows symmetric diffuse abnormal signals in swollen white matter, with or without subcortical cysts in fronto-parietal and anterior-temporal lobes [1, 3]. The lifespan of patients varies from teens or twenties to forties [4–6]. The morbidity rate is unclear, but it has a higher incidence in Argwals [4]. Diagnosis of MLC is dependent on patients’ clinical features, cranial MRI and genetic analysis. About 163 patients were reported worldwide [1–2, 4, 7–9]. *MLC1* (OMIM 605908) and *GlialCAM* (OMIM 611642) are the two disease-causing genes identified in 2001 and 2011, accounting for 75% and 20% patients, respectively[8, 10]. Until now, more than 84 different mutations in *MLC1* and 21 in *GlialCAM* have been described [7–9, 11,12]. We reported the first Chinese MLC patient in 2006 and 13 Chinese MLC patients analyzed clinically and genetically in 2011[7, 13]. About 16 Chinese patients were clinically diagnosed in which 11 were genetically diagnosed with 15 mutations [7,12, 14–15]. However, clinical and genetic features of Chinese MLC patients have not been discovered completely. In this study, clinical and genetic analysis was performed in 20 MLC patients, identifying 10 novel mutations in *MLC1* and 4 patients with *GlialCAM* mutations. A follow-up study was undertaken first for 27 patients including 19 in this research (Pt16 missed) (Pt, Patient) and 8 from our previous reports [7], elucidating clinical and genetic features for patients with MLC in China.

## Materials and Methods

### Patients

20 patients (Pt1-Pt20) from 19 unrelated families were enrolled at Department of Pediatric Neurology, Pecking University First Hospital since 2007 to 2015 based on the criteria proposed by van der Knaap et al. (http://www.genetests.org). Pt19 was from our previous case report in Chinese[12]. 12 were male and 8 female. Ages were ranged from 1.42yo (years old) to 13yo, with a median age of 5.42yo. Pt9 and Pt10 were identical twins. Their clinical data was collected, such as age, sex, onset-symptom, cranial MRI et al.

The study was approved by Ethics committee of Pecking University First Hospital, and written informed consents were obtained from all patients or their legal guardians.

### Genetic analysis

DNA preparation, amplifications and sequencings of *MLC1* and *GlialCAM* were performed as before[7, 12]. *MLC1* sequencing was analyzed for Pt1-Pt20 and *GlialCAM* sequencing for Pt16-Pt20. *MLC1* dosage analysis was held by Multiple Ligation-dependent Probe Amplification (MLPA) with P107 kit (MRC-Holland, Amsterdam, the Netherlands) for Pt13-Pt15 and Pt20.

The splicing mutation c.598-2A>C in Pt2 was detected by reverse transcription-polymerase chain reaction (RT-PCR). Fresh peripheral venous blood was collected from him and his parents. RNA extraction and reverse transcription were performed separately with TRI reagent BD (Catalog number T3809) and SuperScriptTM Ⅲ Reverse Transcriptase(Cat.No.18080-093) as the manufacture’ recommendations. Sequences analysis was performed using the *MLC1* cDNA as the reference.

Haplotype analysis of Pt14-Pt15 and their parents was performed as reported previously [16].

### Follow-up methods

27 patients (Pt1-Pt28) were enrolled into the follow-up study except Pt16 missed, including 8(Pt21-Pt28) from our previous study. Pt21-Pt28, with siblings of Pt24-Pt25 and Pt27-Pt28, described as Pt1-Pt3, Pt6-Pt8, Pt10-Pt11 in our previous report. 15 were male and 12 female. Follow-up research was carried on by telephone survey, head circumference (HC), the information of subsequent motor and cognitive development and deterioration, cranial MRI, head trauma histories and other neurological symptoms including epileptic seizures were collected mainly. Motor ability was evaluated with the Gross Motor Function Classification System (GMFCS) for cerebral palsy, Chinese version [17–18]. Classification from GⅠ to GⅤ was presented mildly to severely impaired motor ability according to their motor function and age. Patients were divided into 5 groups (Group1-5) depend on ages consist of 0–2ys, 2–4ys, 4–6ys, 6–12ys and 12–18ys, respectively. Cognitive evaluation was assessed by their word expression and performance at school. Classification from CⅠto CⅢ expressed different performance from high to low level in school according to their scores. CⅠto CⅢ was normal level, mild impaired and moderately impaired.

## Results

### Clinical findings

20 patients were identified and evaluated clinically in this study (Table 1). Onset age was ranged from birth to 5 months old (mo), with a median of 2mo.

**Table 1:** Clinical features of 20 patients with MLC.

Pt	Sex	Age ^onset^	Age*	Age	HC (cm)	HCtr	IS	IW	WE	G*	PS	EPS	Ata-xia	FH	MRI		
															Age	WMA	Cysts
**1**	**F**	**0m**	**1y**	**9y2m**	**55**	**3m**	**7m**	**1y6m**	**14m**	**Ⅱ**	**-**	**-**	**-**	**-**	**1y**	**+**	**+**
**2**	**M**	**5m**	**5y3m**	**13y**	**54.5**	**3m**	**8m**	**1y6m**	**1y**	**Ⅰ**	**-**	**+**	**-**	**+**	**5y**	**+**	**+**
**3**	**M**	**4m**	**3y4m**	**10y7m**	**55.5**	**3m**	**12m**	**1y6m**	**1y**	**Ⅰ**	**+**	**-**	**+**	**-**	**3y4m**	**+**	**+**
**4**	**M**	**0m**	**3y7m**	**10y1m**	**57.5**	**3m**	**8m**	**1y5m**	**3y**	**Ⅰ**	**-**	**-**	**-**	**-**	**3y**	**+**	**+**
**5**	**F**	**2m**	**10m9d**	**5y9m**	**50.5**	**3m**	**6m**	**2y**	**11m**	**Ⅰ**	**-**	**-**	**-**	**-**	**10m**	**+**	**+**
**6**	**F**	**0m**	**1y9m**	**5y7m**	**52**	**3m**	**7m**	**1y8m**	**1y**	**Ⅰ**	**-**	**-**	**-**	**-**	**1y9m**	**+**	**+**
**7**	**M**	**5m**	**2y1m**	**4y6m**	**57**	**3m**	**6m**	**2y**	**1y7m**	**Ⅰ**	**-**	**+**	**-**	**-**	**1y8m**	**+**	**+**
**8**	**M**	**2m**	**1y5m**	**3y2m**	**56**	**3m**	**7m**	**1y3m**	**1y**	**Ⅰ**	**-**	**+**	**-**	**-**	**1y**	**+**	**+**
**9**	**F**	**5m**	**1y4m**	**3y**	**52.5**	**3m**	**7m**	**3y**	**1y**	**Ⅰ**	**-**	**+**	**-**	**+**	**1y4m**	**+**	**+**
**10**	**F**	**5m**	**1y4m**	**3y**	**/**	**3m**	**7m**	**3y**	**1y**	**Ⅰ**	**-**	**+**	**-**	**+**	**1y4m**	**+**	**+**
**11**	**M**	**0m**	**11m**	**1y5m**	**51.5**	**3m**	**6m**	**1y3m**	**11m**	**Ⅰ**	**-**	**-**	**-**	**-**	**5m, 8m**	**+**	**+**
**12**	**M**	**2m**	**1y8m**	**2y1m**	**55**	**2m**	**8m**	**1y8m**	**8m**	**Ⅱ**	**-**	**+**	**-**	**-**	**1y4m**	**+**	**+**
**13**	**M**	**0m**	**2y2m**	**4y1m**	**57**	**3m**	**11m**	**1y6m**	**1y**	**Ⅰ**	**+**	**-**	**+**	**-**	**1y8m**	**+**	**+**
**14**	**F**	**0m**	**3y6m**	**5y8m**	**60**	**3m**	**7m**	**1y6m**	**10m**	**Ⅱ**	**+**	**-**	**-**	**-**	**3y2m**	**+**	**+**
**15**	**F**	**2m**	**9m**	**2y5m**	**50**	**NA**	**7m**	**1y8m**	**1y**	**Ⅰ**	**-**	**-**	**-**	**-**	**9m**	**+**	**+**
**16**	**M**	**5m**	**9m**	**6y3m**	**50**	**3m**	**7m**	**NA**	**NA**	**Ⅰ**	**-**	**+**	**-**	**-**	**9m**	**+**	**+**
**17**	**M**	**0m**	**1y5m**	**7y6m**	**53**	**7m**	**8m**	**1y1m**	**13m**	**Ⅰ**	**-**	**-**	**-**	**+**	**1y3m**	**+**	**+**
**18**	**M**	**4m**	**9m3d**	**4y3m**	**50**	**4m**	**7m**	**1y9m**	**1y6m**	**Ⅰ**	**-**	**+**	**-**	**-**	**8m**	**+**	**+**
**19**	**F**	**4m**	**5y**	**12y6m**	**55**	**4m**	**8m**	**2y**	**1y2m**	**Ⅱ**	**-**	**-**	**-**	**-**	**5y**	**+**	**+**
**20**	**M**	**0m**	**10.5m**	**5y3m**	**52**	**4m**	**10m**	**1y6m**	**2y**	**Ⅰ**	**+**	**-**	**-**	**-**	**10.5m**	**+**	**+**

Abbreviations: F, female; Age^onset^, onset age; Age*, age at first diagnosis; Age, age at the third follow-up study; M, male; y, year; m, month; HC, Head circumference(cm); HCtr, Head Control; IS, independent sitting; IW, independent walking; WE, word expression; G*, gross motor function classification system at first diagnosis; PS, pyramidal signs; EPS, extralpyramidal signs; FH, family history; MRI, magnetic resonance imaging; WMA, white matter abnormal signals;+,positive;-,negative

**Fig 1:**
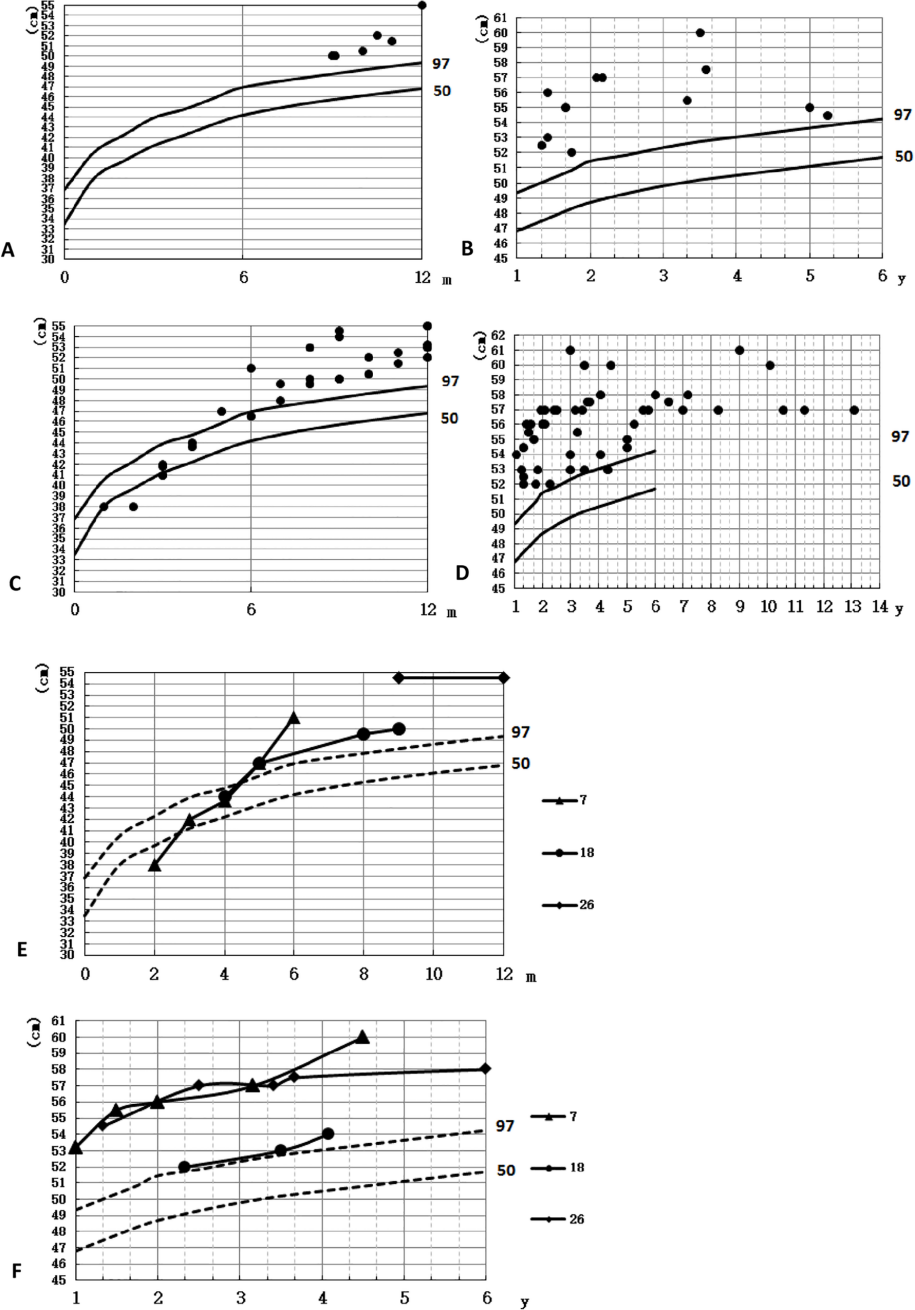
The head circumferences of patients. y, year; m, month. (A-B) HC of 20 patients at first diagnosis; (C-D) HC of patients at the follow-up study; (E-F) HC growth curve of Pt7, Pt18 and Pt26.

**Fig 2:**
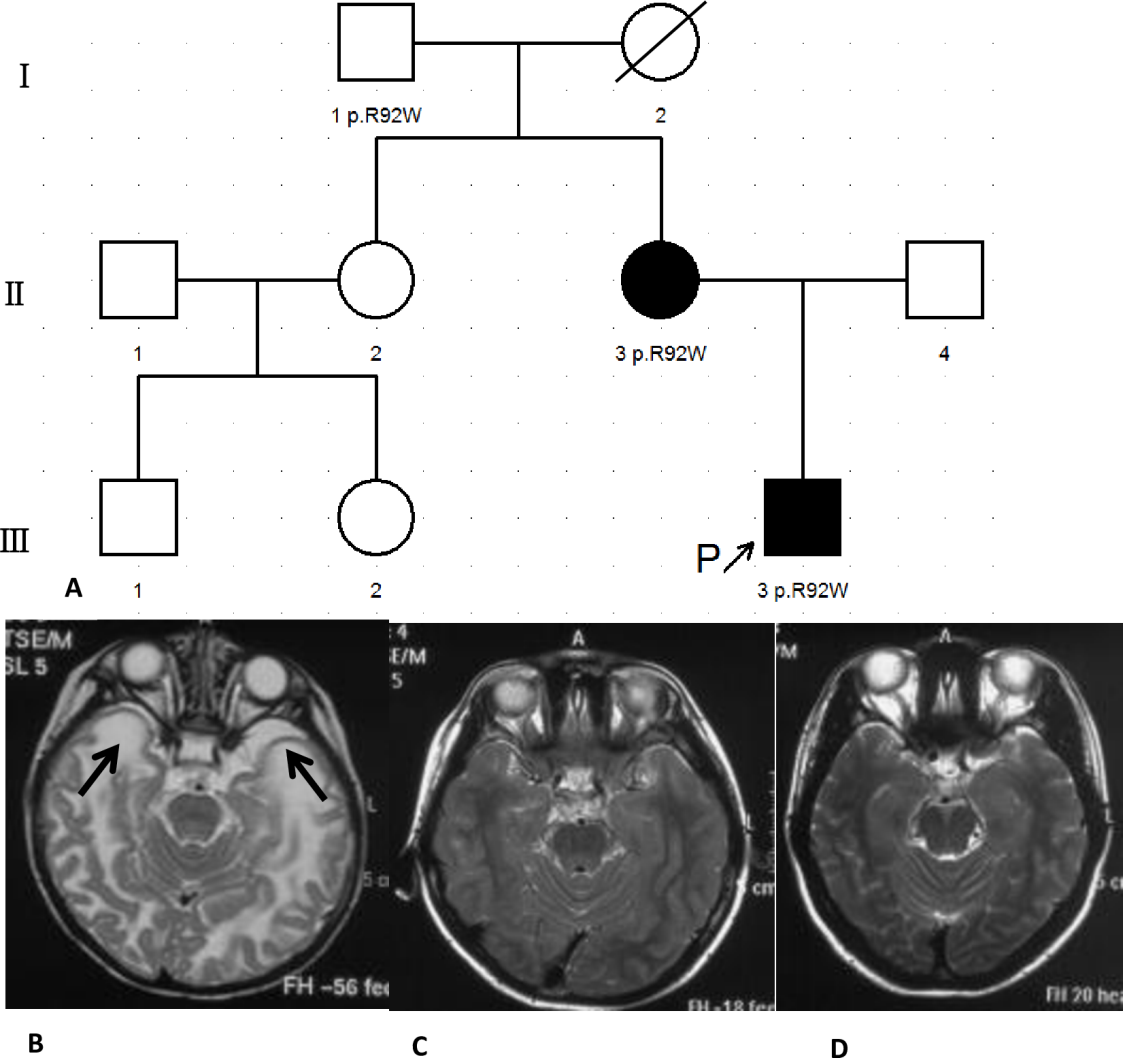
Genogram and cranial MRI of Pt17 and his mother. (A) Genogram of Pt17. (B) MRI of Pt17 at 7mo. Abnormal signals in the white matter with subcortical cysts in the temporal lobes (Arrows). (C) MRI of Pt17 at 7.67yo. Normal signals in the white matter without subcortical cysts in the temporal lobes. (D) MRI of his mother at 33yo. Normal signals in the white matter without subcortical cysts in the temporal lobes.

Macrocephaly was found in total 20 patients and their HC were beyond the 95^th^ percentile of the control (Fig 1A and 1B). Out of 20, 17(85%) patients showed motor development delay, 3(Pt8, Pt11, Pt16) (15%) patients had normal development. According to the motor ability assessment(GMFCS), 16(80%) patients were GⅠ, 4(20%) GⅡ. 19 of 20 were assessed with cognitive development, among them, 4(Pt4, Pt7, Pt18, Pt20) (20%) patients were found speech development delay and 15(75%) with normal speech development, and 1(Pt16) (5%) was too young to evaluate. Generalized tonic-clonic seizures without head trauma or fever were appeared in Pt4. Pt2’s elder sister had similar symptoms with him and died at her 5yo due to severe infection. Pt9 and Pt10 were identical twins with similar phenotype. Macrocephaly without motor or cognitive development delay was found in Pt17’s mother, and neither macrocephaly nor development delay was described from his grandfather. Typical abnormal signals in diffuse swollen white matter and subcortical cysts in temporal, fronto-parietal or parieto-occipital lobes were found in all 20 patients (Fig 2B). No asphyxia at delivery was found, birth weight and body length were normal in 20 patients. Their parents were not consanguineous. All enrolled 20(100%) subjects were clinically diagnosed with MLC.

### Genetic findings

Genetic analysis was performed in 20 patients (Table 2), 19(95%) patients were genetically diagnosed, including 15 patients with *MLC1* mutations and 4 patients with *GlialCAM* mutations. Neither *MLC1* nor *GlialCAM* mutation was found in 1 patient (Pt20) (5%).

**Table 2:** The genotypes in 20 patients with MLC.

Pt	Gene	Mutation	Protein	mRNA	Mutation type	Novel/ Reported	Parental origin
**1**	***MLC1***	**c.772-1G>C**	**Aberrant**	**No**	**Splicing**	**Reported**	**P**
		**c.594delCTCA**	**p.Y198X**	**No**	**Nonsense**	**Reported**	**M**
**2**	***MLC1***	**c.278C>T**	**p.S93L**	**No**	**Missense**	**Reported**	**P**
		**c.598-2A>C**	**Aberrant**	**Exon8 deletion**	**Splicing**	**Reported**	**M**
**3**	***MLC1***	**c.895-1G>A**	**Aberrant**	**No**	**Splicing**	**Reported**	**P**
		**c.824C>A**	**p.A275T**	**No**	**Missense**	**Novel**	**M**
**4**	***MLC1***	**c.772-1G>C**	**Aberrant**	**No**	**Splicing**	**Reported**	**P**
		**c.65G>A**	**p.R22Q**	**No**	**Missense**	**Reported**	**M**
		**c.634G>A**	**p.G212R**	**No**	**Missense**	**Reported**	**M**
**5**	***MLC1***	**c.881C>T**	**p.P294L**	**No**	**Missense**	**Novel**	**P**
		**c.596delCAgt**	**p.S199Cfs220X**	**No**	**Frame shift**	**Novel**	**M**
**6**	***MLC1***	**c.803C>G**	**p.T268R**	**No**	**Missense**	**Novel**	**P**
		**c.353G>T**	**p.T118M**	**No**	**Missense**	**Reported**	**M**
**7**	***MLC1***	**c.803C>G**	**p.T268R**	**No**	**Missense**	**Novel**	**P**
		**c.634G>A**	**p.G212R**	**No**	**Missense**	**Reported**	**M**
**8**	***MLC1***	**c.812T>C**	**p.L271P**	**No**	**Missense**	**Novel**	**P**
		**c.218G>A**	**p.G73E**	**No**	**Missense**	**Reported**	**M**
**9**	***MLC1***	**c.353C>T**	**p.T118M**	**No**	**Missense**	**Reported**	**P**
		**c.606G>C**	**p.E202D**	**No**	**Missense**	**Novel**	**M**
**10**	***MLC1***	**c.353C>T**	**p.T118M**	**No**	**Missense**	**Reported**	**P**
		**c.606G>C**	**p.E202D**	**No**	**Missense**	**Novel**	**M**
**11**	***MLC1***	**c.912_935del**	**p.307_314del**	**No**	**Small deletion**	**Novel**	**P**
		**c.962delG**	**p.G321Afs39X**	**No**	**Small deletion**	**Novel**	**M**
**12**	***MLC1***	**c.929_930insGCTGCT**	**p.309_310insLL**	**No**	**Insertion**	**Reported**	**P**
		**c.184C>T**	**p.L62F**	**No**	**Missense**	**Novel**	**M**
**13**	***MLC1***	**E4-E9deletion**	**-**	**No**	**Deletion**	**Novel**	**P**
		**E4-E9deletion**	**-**	**No**	**Deletion**	**Novel**	**M**
**14**	***MLC1***	**c.772-1G>C**	**Aberrant**	**No**	**Splicing**	**Reported**	***de novo***
		**c.772-1G>C**	**Aberrant**	**No**	**Splicing**	**Reported**	**M**
**15**	***MLC1***	**c.218G>A**	**p.G73E**	**No**	**Missense**	**Reported**	**M**
		**c.218G>A**	**p.G73E**	**No**	**Missense**	**Reported**	**M**
**16**	***MLC1***	**c.95C>T**	**p.A32V**	**No**	**Missense**	**Reported**	**M**
	***GlialCAM***	**c.274C>T**	**p.R92W**	**No**	**Missense**	**Reported**	**M**
**17**	***GlialCAM***	**c.274C>T**	**p.R92W**	**No**	**Missense**	**Reported**	M*
		**c.274C**	**p.R92**	**No**	**No**		**P**
**18**	***GlialCAM***	**c.274C>T**	**p.R92W**	**No**	**Missense**	**Reported**	***de novo***
		**c.274C**	**p.R92**	**No**	**No**		
**19**	***GlialCAM***	**c.395C>A**	**p.T132N**	**No**	**Missense**	**Novel**	**P**
		**c.203A>T**	**p.K68M**	**No**	**Missense**	**Novel**	**M**
**20**	***MLC1***	**c.206C>T**	**p.S69W**	**No**	**Missense**	**Reported**	**P**
		**c.858C>G**	**p.I286M**	**No**	**Missense**	**Novel**	**P**

Abbreviations: MLC, megalencephalic leukoencephalopathy with subcortical cysts; P, paternal; M, maternal; M*, maternal with macrocephaly.

GenBank accession No.NM_015166, position 1 is ‘A’ of the translation initiation codon.

**Fig 3:**
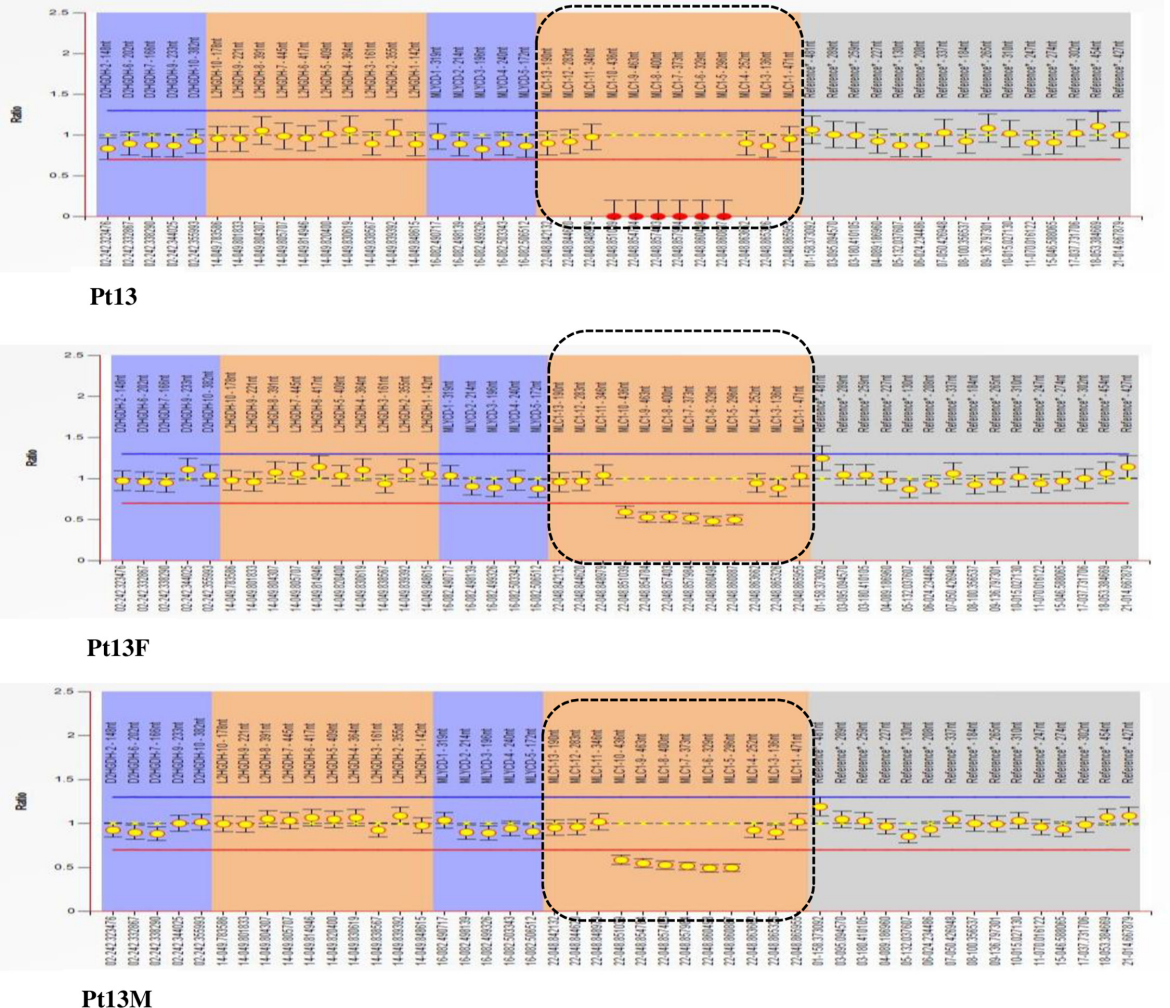
The MLPA results of Pt13. Fragments in the dot box were exons of *MLC1*. Line at the left side represented signal rate. Signal rate between 0.7(red line) and 1.3(blue line) were normal. Signal rate less than 0.7 or greater than 1.3 were deletion or duplication, respectively. It indicated that homozygous deletion from exon4 to exon9 in *MLC1* was detected in Pt13, and his parents were heterozygous deletion (dotted box).

**Fig 4:**
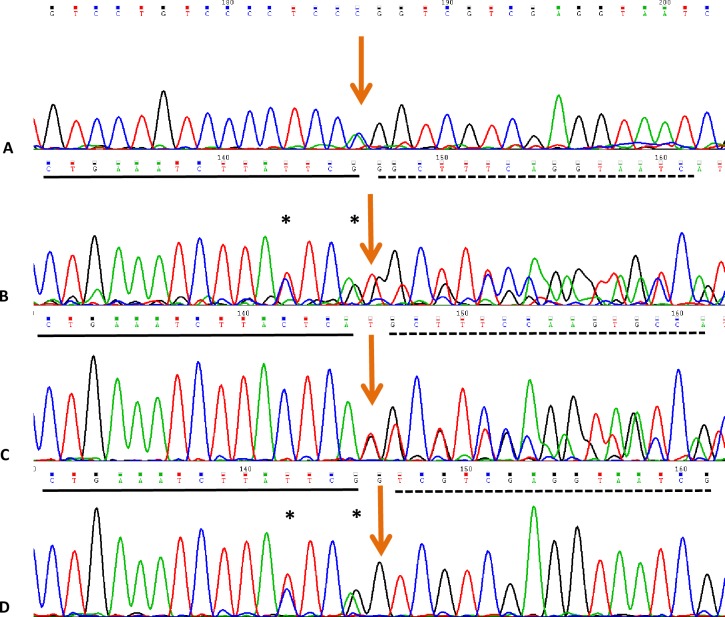
Splicing mutation of c.598-2A>C in *MLC1* leads to skip of exon8. (A) DNA sequence results of Pt2. (B-D) RT-PCR results of Pt2, his mother, his father, respectively. “*” indicated SNPs, arrows meant the mutation site. Exon7 were lined, exon8 and 9 were dotted.

**Fig 5:**
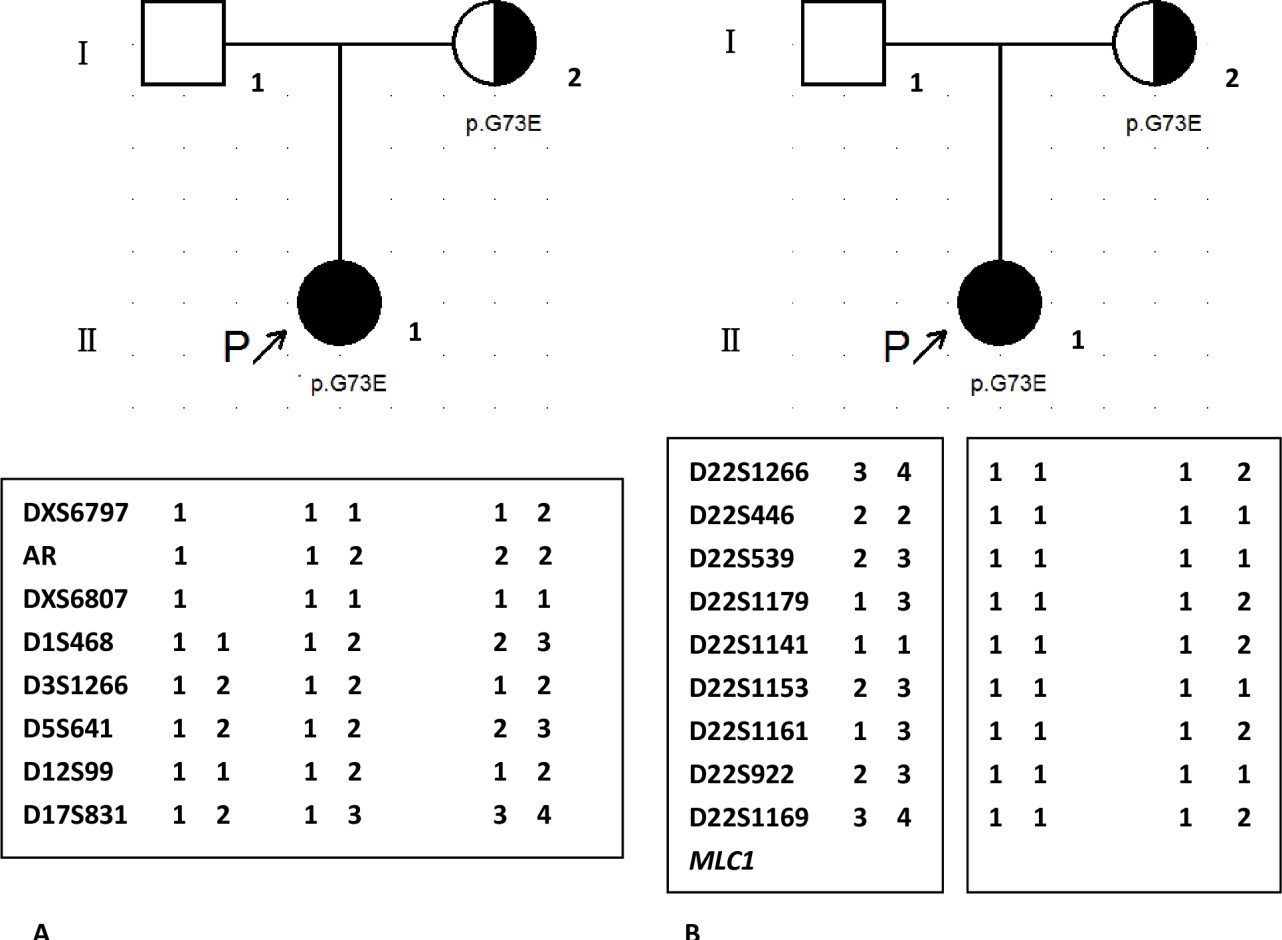
Results of haplotype analysis of Pt15 and her parents. 9 STR located on chromosome 22 were from 22q11.1 to 22q13.33 terminal regions. Alleles were labeled arbitrarily according to size.

*MLC1* mutations were identified in 15 patients (75%). Homozygous or compound heterozygous point mutations consisting of 10 novel mutations including 6 novel missense mutations(c.184C>T(p.L62F), c.606G>C(p.E202D), c.803C>G(p.T268R), c.812T>C(p.L271P), c.824C>A(p.A275T), c.881C>T(p.P294L)), 3 novel small deletion mutations (c.596delCAgt(p.S199Cfs220X), c.912_935del(p.307_314del), c.962delG(p.G321Afs39X)), 1 novel large deletion from exon4 to exon9 (Fig 3), and 11 reported mutations(c.65G>A(p.R22Q), c.95C>T(p.A32V), c.218G>A(p.G73E), c.278C>T(p.S93L), c.353C>T(p.T118M), c.594delCTCA(p.Y198X), c.598-2A>C(splicing site mutation), c.634G>A(p.G212R), c.772-1G>C(splicing site mutation), c.895-1G>A(splicing site mutation), c.929_930insGCTGCT(p.309_310insLL)) were found. Three heterozygous mutations were identified in Pt4, in which one allele (c.772-1G>C) came from her father and two sites (p.R22Q, p.G212R) in one allele were inherited from her mother. For the splicing mutation (c.598-2A>C) in Pt2, skip of exon8 was found by cDNA test (Fig 4). Homozygous mutations (c.772-1G>C; p.G73E) were found in Pt14 and Pt15, respectively. Heterozygous variation was showed in their mothers, and wild type in their fathers. Dosage analysis of them was normal. Chromosome22 were inherited from her parents in Pt14 (Data not shown), however two chromosome22 were maternal origin in Pt15 by short tandem repeats (STR) analysis (Fig 5). Those results might indicate a *de novo* point mutation (c.772-1G>C) in Pt14 or her father mosaic and identify maternal uniparental disomy (UPD) in Pt15.

**Fig 6:**
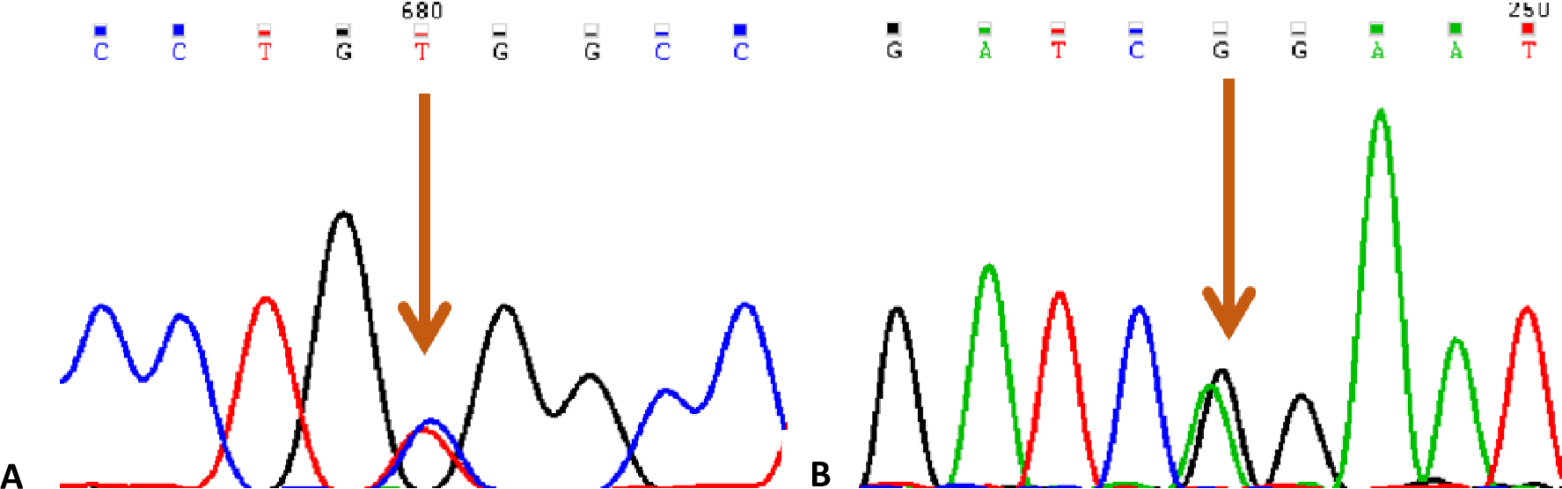
Genetic results of Pt16. (A) Heterozygous variation of c.95C>T (p.A32V) in *MLC1*; (B) Heterozygous variation of c.274C>T (p.R92W) in *GlialCAM*.

*GlialCAM* mutations with 3 (c.203C>T (p.K68M), c.274C>T (p.R92W), c.395C>A (p.T132N)) mutations were detected in Pt16-Pt19. Both heterozygous (p.A32V) variation in *MLC1* and heterozygous (p.R92W) mutation in *GlialCAM* were detected in Pt16 (Fig 6).

### Follow-up study findings

27 patients including 19 in this study (Pt16 missed) and 8(Pt21-Pt28) in our previous report were enrolled in the follow-up study (Table 3) [7]. Among them, 13 achieved total three times follow-up study. 16, 19, 22 patients participated the first, second, third study, respectively, with a median age of 5.42yo, 7yo, 5.63yo and a median clinical course of 5.46 years, 7 years and 5.42 years.

**Table 3:** Summaries of clinical features.

Pt	Sex	At first diagnosis			1^st^ follow-up study			2^nd^ follow-up study			3^rd^ follow-up study			Sz	LC	MR
		Age	T(y)	G	Age	T(y)	G	Age	T(y)	G	Age	T(y)	G			
**1**	**F**	**1y**	**1**	**Ⅱ**	**5y7m**	**5.67**	**Ⅱ**	**7y**	**7**	**Ⅱ**	**/**	**/**	**/**	**-**	**-**	**-**
**2**	**M**	**5y3m**	**4.67**	**Ⅰ**	**9y7m**	**9.17**	**Ⅲ**	**11y**	**10.58**	**Ⅲ**	**13y**	**12.58**	**Ⅲ**	**+**	**+**	+*
**3**	**M**	**3y4m**	**3**	**Ⅰ**	**7y1m**	**6.67**	**Ⅱ**	**8y6m**	**7.08**	**Ⅱ**	**10y7m**	**10.25**	**Ⅱ**	**-**	**-**	**-**
**4**	**M**	**3y7m**	**3.58**	**Ⅰ**	**6y7m**	**6.58**	**Ⅰ**	**8y**	**8**	**Ⅰ**	**10y1m**	**10.08**	**Ⅰ**	**+**	**-**	**+**
**5**	**F**	**10m**	**0.67**	**Ⅰ**	**2y3m**	**2.58**	**Ⅰ**	**3y8m**	**3**	**Ⅰ**	**5y9m**	**5.08**	**Ⅰ**	**-**	**+**	**+**
**6**	**F**	**1y9m**	**1.75**	**Ⅰ**	**2y2m**	**2.17**	**Ⅰ**	**3y7m**	**3.58**	**Ⅰ**	**5y7m**	**5.58**	**Ⅰ**	**-**	**-**	**-**
**7**	**M**	**2y1m**	**1.67**	**Ⅰ**	**N**	**N**	**N**	**2y4m**	**2.25**	**Ⅰ**	**4y6m**	**4.08**	**Ⅰ**	**+**	**-**	**-**
**8**	**M**	**1y5m**	**1.25**	**Ⅰ**	**N**	**N**	**N**	**N**	**N**	**N**	**3y2m**	**3**	**Ⅰ**	**-**	**-**	**+**
**9**	**F**	**1y4m**	**0.92**	**Ⅰ**	**N**	**N**	**N**	**N**	**N**	**N**	**3y**	**2.58**	**Ⅰ**	**-**	**-**	**+**
**10**	**F**	**1y4m**	**0.92**	**Ⅰ**	**N**	**N**	**N**	**N**	**N**	**N**	**3y**	**2.58**	**Ⅰ**	**-**	**-**	**+**
**11**	**M**	**11m**	**0.92**	**Ⅰ**	**N**	**N**	**N**	**N**	**N**	**N**	**1y5m**	**1.42**	**Ⅰ**	**-**	**-**	**-**
**12**	**M**	**1y8m**	**1.5**	**Ⅱ**	**N**	**N**	**N**	**N**	**N**	**N**	**2y1m**	**1.92**	**Ⅰ**	**+**	**-**	**-**
**13**	**M**	**2y2m**	**2.17**	**Ⅰ**	**N**	**N**	**N**	**N**	**N**	**N**	**4y1m**	**3.92**	**Ⅰ**	**+**	**-**	**+**
**14**	**F**	**3y6m**	**3.5**	**Ⅱ**	**N**	**N**	**N**	**N**	**N**	**N**	**5y8m**	**5.67**	**Ⅰ**	**+**	**-**	**+**
**15**	**F**	**9m**	**0.58**	**Ⅰ**	**N**	**N**	**N**	**N**	**N**	**N**	**2y5m**	**2.25**	**Ⅰ**	**+**	**-**	**+**
**16**	**M**	**9m**	**0.33**	**Ⅰ**	**/**	**/**	**/**	**/**	**/**	**/**	**/**	**/**	**/**	**-**	**-**	**-**
**17**	**M**	**1y5m**	**1.42**	**Ⅰ**	**4y**	**3.67**	**Ⅰ**	**5y5m**	**5.08**	**Ⅰ**	**7y6m**	**7.5**	**NOR**	**-**	**-**	**-**
**18**	**M**	**9m**	**0.42**	**Ⅰ**	**1y**	**0.58**	**Ⅰ**	**2y3m**	**1.83**	**Ⅰ**	**4y3m**	**3.92**	**NOR**	**-**	**-**	**-**
**19**	**F**	**5y**	**4.67**	**Ⅱ**	**9y**	**8.57**	**Ⅱ**	**10.5y**	**10.17**	**Ⅱ**	**12y6m**	**12.17**	**Ⅱ**	**-**	**+**	+*
**20**	**M**	**10m**	**0.83**	**Ⅰ**	**1y9m**	**1.75**	**Ⅰ**	**3y2m**	**3.17**	**Ⅰ**	**5y3m**	**5.25**	**Ⅰ**	**+**	**-**	**-**
**21**	**M**	**7y4m**	**6.67**	**Ⅱ**	**13y**	**12.17**	**Ⅱ**	**14.5y**	**13.75**	**Ⅱ**	**16y7m**	**15.92**	**Ⅱ**	**+**	**-**	**+**
**22**	**F**	**10m**	**0.42**	**Ⅰ**	**6y5m**	**6**	**Ⅱ**	**7.8y**	**7.42**	**Ⅱ**	**10y**	**9.67**	**NOR**	**-**	**-**	**-**
**23**	**F**	**1y1m**	**1.08**	**Ⅱ**	**5y3m**	**5.25**	**Ⅲ**	**6y6m**	**6.5**	**Ⅴ**	**8y7m**	**8.58**	**Ⅴ**	**+**	**-**	+*
**24**	**M**	**8m**	**0.25**	**Ⅱ**	**4y3m**	**3.83**	**Ⅱ**	**5y8m**	**5.25**	**Ⅱ**	**/**	**/**	**/**	**-**	**-**	**+**
**25**	**F**	**9y**	**8.58**	**Ⅰ**	**12.5y**	**12.08**	**Ⅰ**	**14y**	**13.58**	**Ⅰ**	**/**	**/**	**/**	**-**	**-**	**-**
**26**	**M**	**1y3m**	**1.25**	**Ⅰ**	**3y7m**	**3.58**	**Ⅱ**	**4y**	**4**	**Ⅱ**	**7y2m**	**7.17**	**Ⅲ**	**+**	**-**	+*
**27**	**F**	**11.3y**	**11.3**	**Ⅲ**	**N**	**N**	**N**	**15.5y**	**15.58**	**Ⅲ**	**/**	**/**	**/**	**-**	**-**	**-**
**28**	**M**	**9y**	**9**	**Ⅲ**	**N**	**N**	**N**	**13.25**	**13.25**	**Ⅳ**	**/**	**/**	**/**	**-**	**-**	+*

Abbreviations: F, female; M, male; y, year; m, month; T, time of disease course; G, gross motor function classification system; /, lost to follow up; N, not enrolled in the research; Sz, seizures; LC, loss of consciousness; MR, motor retrogress;-,negative;+,positive; NOR, normal; +*,positive, retrogressed and not recovered

Macrocephaly was appeared at birth or at initial months (Table 4). HC increased rapidly during the first year, and maintained beyond the 95^th^ percentile, but it grew slowly with growing up (Fig 1C and 1D). HC of patients older than 5yo was ranged from 56cm to 61cm, with a median of 57cm. HC growth curve of Pt7, Pt18 and Pt26 showed that Pt7 suffered two fast increasing periods of 0 to 2yo and 3.17yo to 4.5yo; HC of Pt18 and Pt26 grew rapidly before 1yo without a second obvious fast increasing period (Fig 1E and 1F). For motor ability results out of 16 patients at the first follow-up study, 7(43.8%) were GⅠ, 7(43.8%) GⅡ, 2(12.5%) GⅢ; of 19 participated the second follow-up study, 8(42.1%) were GⅠ, 7(36.8%) GⅡ, 2(10.5%) GⅢ, 1(5.3%) GⅣ,1(5.3%) GⅤ. Total 22 patients at the third follow-up study, 16(72.7%) were GⅠ, 3(13.6%) GⅡ, 2(9.1%) GⅢ, 1(4.5%) GⅤ. Out of 27 patients, motor deterioration was found in 5(Pt2, Pt19, Pt23, Pt26, Pt28) (18.5%) patients at their 8, 11, 2.5, 6.5 and 10yo, respectively. Pt2 suffered a traffic accident at 8yo, falling in coma for 1 week, then became wheel-dependent. Loss of walking independently occurred in Pt9 and Pt26, and paralysis, dysarthria and dysphagia were found in Pt23. The remaining 22 patients had a stable condition or progresses gradually. Motor ability of Pt17, Pt18 and Pt22 was normal.

**Table 4:** Head Circumference in the follow-up study.

**Pt**	**1**	**2**	**3**		**4**	**5**	**6**	**7**	**7**	**8**	**9**
**MC onset**	**Birth**	**5m**	**4**		**Birth**	**2**	**Birth**	**5**		**2**	**5m**
**HC-IP(m)**	**0–12**	**5–12**	**4–12**		**0–12**	**2–10**	**0–12**	**5–12**		**4–7**	**0–12**
**HC/cm(Age)**	**55(1y)**	**54.5(5y)**	**55.5(3y3m)**		**57.5(3y7m)**	**50.5(10m)**	**52(1y9m)**	**38(2m)**	**53.2(12m)**	**56(1y5m)**	**41(3m9d)**
		**57(13y)**	**57(8y3m)**		**60(10y1m)**	**53(4y4m)**	**56.8(5y7m)**	**42(3m)**	**55.5(18m)**	**61(3y)**	**52.5(1y4m)**
			**57(10y7m)**			**57(5y9m)**		**43.6(4m)**	**56(2y)**		**53(3y)**
								**47(5m)**	**57(3y2m)**		
								**51(6m)**	**60(4y6m)**		
**Pt**	**10**	**11**	**12**	**12**	**13**	**14**	**15**	**16**	**17**	**18**	**18**
**MC onset**	**5m**	**Birth**	**2m**		**Birth**	**Birth**	**2m**	**5m**	**Birth**	**4m**	
**HC-IP(m)**	**0–12**	**3–7**	**0–12**		**0–7**	**0–12**	**2–9**	**5–9**	**0–12**	**0–12**	
**HC/cm(Age)**	**41(3m9)**	**49.5(7m)**	**38(1m)**	**52(1y)**	**56 (1y7m)**	**52.5 (11m)**	**48(7m)**	**50 (9m)**	**53 (1y)**	**44 (4m)**	**52 (2y3m)**
	**54(3y)**	**51.5 (11m)**	**41.8 (3m)**	**55 (1y8m)**	**57 (2y1m)**	**60 (3y6m)**	**50(9m)**		**53(1y3m)**	**47 (5m)**	**53 (3y6m)**
		**52 (1y4m)**	**46.5 (6m)**	**56(2y)**	**58 (4y1m)**		**57(1y11m)**		**57.5(6y6m)**	**49.5(8m)**	**54 (4y1m)**
			**50(8m)**	**56 (2y1m)**			**57(2y5m)**			**50 (9m)**	
**Pt**	**19**	**20**	**21**	**22**	**23**	**24**	**25**	**26**	**26**	**27**	**28**
**MC onset**	**4m**	**Birth**	**8m**	**4m**	**Birth**	**5m**	**5m**	**Birth**		**Birth**	**Birth**
**HC-IP(m)**	**3–12**	**0–12**	**8–11**	**4–12**	**3–30**	**5–12**	**5–12**	**3–9**		**0–12**	**0–12**
**HC/cm(Age)**	**53(1y10)**	**52(10m)**	**57(7y)**	**54(9m)**	**54(13m)**	**53(8m)**	**55(1y)**	**54.5(9m)**	**57.5(3y8m)**	**57(11y4m)**	**61(9y)**
	**55(5y)**	**56(5y3m)**						**54.5(1y4m)**	**58(6y)**		
								**57(2y6m)**	**58(7y2m)**		
								**57(3y5m)**			

Abbreviations: MC, macrocephaly; HC-IP, head circumference increasing period; HC, head circumference; y, year; m, month

Cognitive ability was evaluated for 22 out of 27 except Pt1, Pt24, Pt25, Pt27, Pt28. Among 22, 6(Pt7, Pt9-Pt12, Pt15) were too young to go to school, but their parents considered their mental ability normal; 5(Pt2, Pt5, Pt14, Pt23, Pt26) didn’t go to school because of motor disability, 11 accepted routine school education used for the following cognitive classification evaluation. Out of 11, 3(Pt17, Pt18 and Pt22)(27.3%) were CⅠ, 4(Pt2, Pt6, Pt13, Pt20)(36.4%) CⅡ, and 4(Pt3, Pt4, Pt19, Pt21) (36.4%) CⅢ.

**Fig 7:**
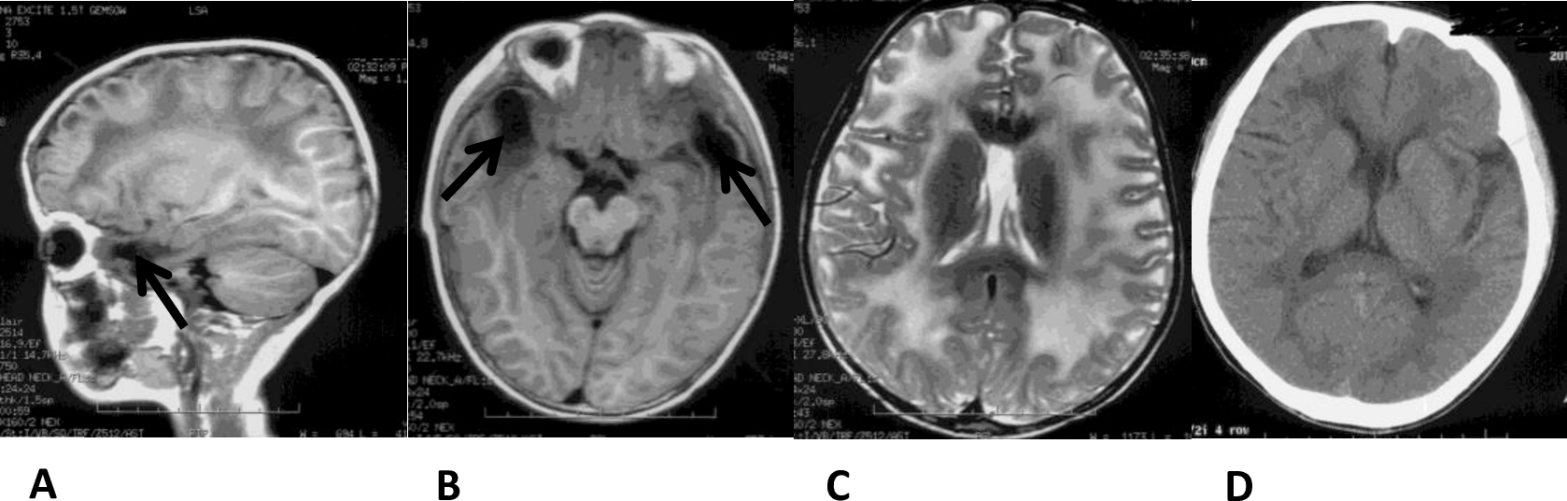
CT and MRI of Pt2. (A-C): cranial MRI at his 4.08yo. Diffuse and abnormal swollen white-matter signals and subcortical cysts in the temporal lobes (arrows); (D) CT at his 12.75yo indicated no changes in the white matter and subcortical cysts.

Cranial MRI/CT examination for more than 2 times were took in Pt2 and Pt17. Four times for Pt2 was re-examined after the accident, and no obvious changes were found (Fig 7). Classic abnormalities in cranial MRI were detected in Pt17 at 7mo, but it improved obviously even both abnormal signals in white matter and cysts had disappeared at 7.67yo (Fig 2).

Out of 22 patients from the third follow-up study, 10(Pt2, Pt4, Pt7, Pt12-Pt15, Pt20, Pt21, Pt26)(45.5%) patients with seizures and 3(Pt2, Pt5, Pt19)(11.1%) patients with unconsciousness were found after minor head trauma. 4(Pt14, Pt15, Pt23, Pt26)(18.2%) patients with seizures and 0 with unconsciousness were found after fever. Transient motor retrogress occured in 10(Pt2, Pt5, Pt8-Pt10, Pt13-Pt15, Pt21, Pt26)(45.5%) and 2(Pt4, Pt26) patients after minor head trauma or fever, respectively, mainly being hypotonic and lost to walk independently, taking 1 to 2 weeks to recover for most patients but about 2 months for Pt9 and Pt10. Pt4 and Pt14 were treated with Topiramate and Levetiracetam, respectively, and no seizures were appeared even after head trauma after then. Ataxia was found in 10 patients.

## Discussion

Megalencephalic leukoencephalopathy with subcortical cysts is a neurodegenerative disease caused by mutations in *MLC1* or *GlialCAM* [8, 10]. Classic phenotype is characterized by infant-onset macrocephaly, different degrees of motor development delay, seizures, spasticity, motor deterioration, pyramidal and extrapyramidal dysfunction with abnormal signals in white matter and subcortical cysts in an autosomal recessive mutations in *MLC1* or *GlialCAM* [8, 10]; the atypical improving phenotype has a similar phenotype in the initial stage, but would improve or even normalize with dominant mutations in *GlialCAM* [8–9]. In this research, 20 patients met the criteria of MLC with clinical and typical cranial MRI features similar to the previous reports [1, 7–9, 11].

Macrocephaly was the initial symptoms of 20 patients, with onset before 5mo. But most of them came to hospital because of development delay or seizures after minor head trauma. Motor development delay and cognitive impairment were found in 85% (17/20) and in 20% (4/20) patients, respectively. Diffuse abnormal signals in swollen white matter and subcortical cysts were detected in 100% (20/20) patients. Clinical improvements were found in 2 (Pt17, Pt18) patients, and recovery in cranial MRI was appeared in Pt17. Total 20 patients were divided into 17 classic phenotype, 2(Pt17, Pt18) atypical improving phenotype, and 1(Pt16 missed) unclassified.

**Fig 8:**
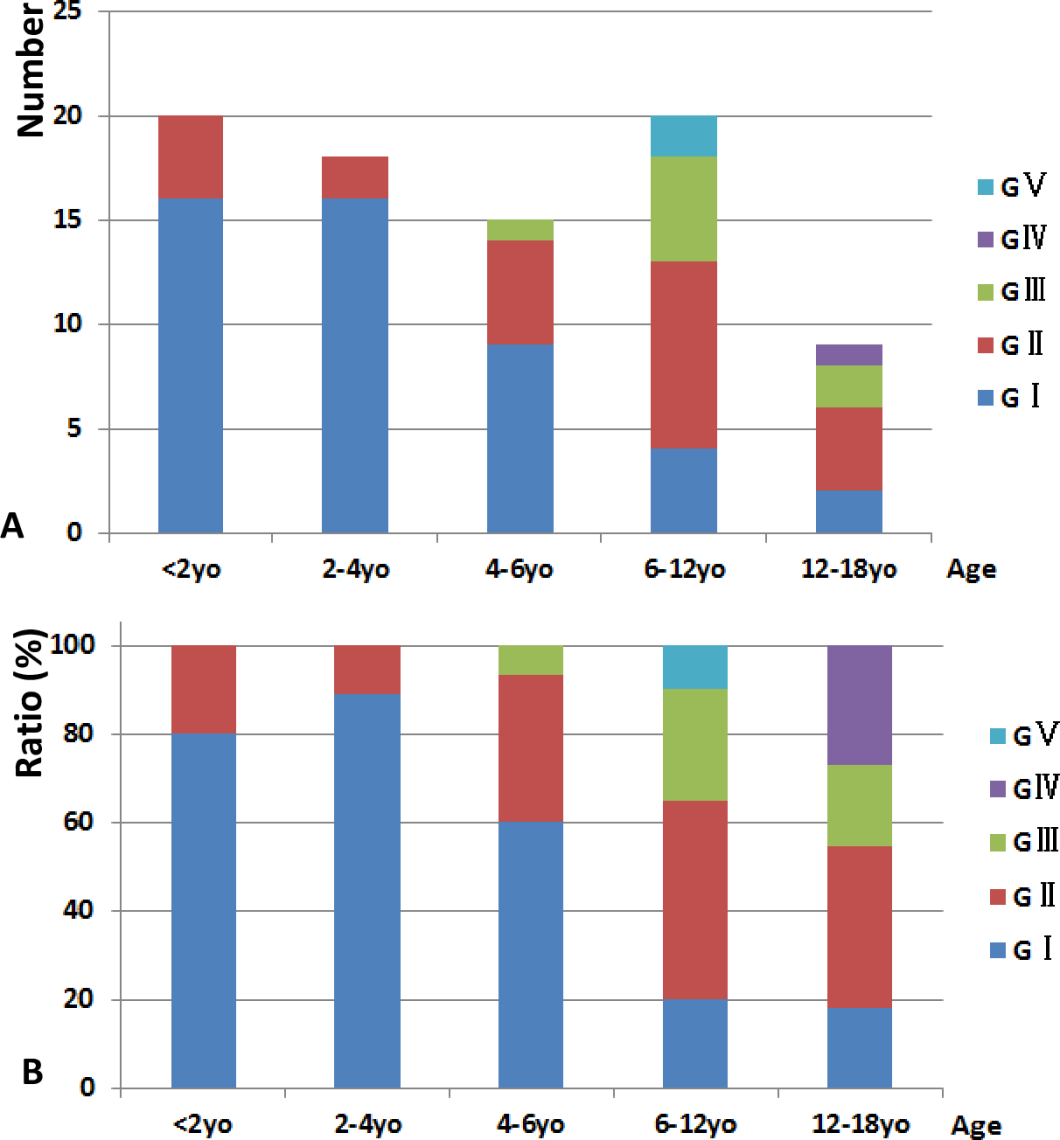
Classification of motor ability. (A)Patients numbers in each motor ability group; (B) Ratio of each levels of motor ability in groups of age.

For all the 27 patients enrolled in the follow-up study, HC increased rapidly during the first year similar with previous reports [1, 7]. Interestingly, we found another fast increasing period between 3.17yo and 4.5yo in 1 (Pt7) patient (Fig 1E and 1F), which was first discovered. Motor ability of MLC patients was age-relative: GⅠor GⅡoccurred in patients under 4yo while GⅢ to GⅤin patients only appeared in patients older than 4yo (Fig 8). Ratio of GⅠ increased from Group1 to Group2 and sharply decreased after then. Ratio of GⅡ decreased from Group1 to Group2 and increased until Group4 but decreased in Group5 since patients of GⅢ to GⅤ increasing. Patients of GⅢ were older than 4yo, and ratio of it increased from Group3 to Group4 and decreased in Group5 since patients of GⅣ increasing. Pt23 was paralyzed with GⅤ since 6.5yo and Pt28 was GⅣ since 13yo. It revealed that motor development of Chinese MLC patients was delayed but they obtained a developing period until 4yo with fewer differences between them and the unaffected, they were more backward than the normal individuals after 6yo. But more samples were needed to be analyzed to confirm it. Motor deterioration between 2.5yo to 11yo was found in 5 (18.5%) patients, including 1(Pt2) due to a traffic accident, showing deterioration onset was earlier than previous reports [19] but later than that Kariminejad reported [11]. Cognitive ability of 27 was relatively spared. Patients could perform as the unaffected before Grade2, however intellectual disability was obvious after Grade2 in primary school. Cognitive deterioration was found in 2 (Pt19, Pt23) (7.4%) patients. Out of 22 patients in the third follow-up study, seizures occurred in 11(50%) patients after minor head trauma or fever which was less than the reported rate of 60% [20]. Unconsciousness was occurred in 3(13.6%) patients only after head trauma for several minutes to hours except Pt2 for 8 weeks. Otherwise Bugiani M et al reported a boy lost consciousness over 4mo after a minor head trauma [21]. Rate of transient motor retrogress was found in 11(50%) patients after minor head trauma or fever. Neither deterioration nor seizures were found in atypical improving MLC patients (Pt17, Pt18). Interestingly, rate of seizures (45.5%), transient motor retrogress (45.5%) and unconsciousness(13.6%) after minor head trauma was much higher than that after fever(18.2%, 9.1%, 0%, respectively). In our research, seizures, unconsciousness and transient motor retrogress were more effective after minor head trauma than after fever.

In all 20 patients, 19(Pt1-Pt19)(95%) were genetically diagnosed including 15(75%) with *MLC1* mutations and 4(20%) with *GlialCAM* mutations, similar with the other researches[2, 8], and 1(Pt20) (5%) was not found mutations of *MLC1* or *GlialCAM*. Up to date, more than 84 MLC-relevant mutations of *MLC1* and 21 of *GlialCAM* have been reported from various ethnic backgrounds[2, 7–9, 11–12], 11 reported and 10 novel mutations were identified in *MLC1*, and 3 mutations were found in *GlialCAM* in this study, which greatly expanded the mutation spectrum. All the novel mutations were neither found in 200 control alleles nor in the 1000 genome database (http://browser.1000genomes.org/index.html).

15(75%) patients were identified with mutations in *MLC1* with 21 mutations including 11 reported and 10 novel mutations. 7 novel mutations (p.L62F, p.A275T, p.S199Cfs220X, p.E202D, p.T268R, p.L271P, p.307_314del,) of *MLC1* occurred in transmembrane domains in the MLC1 protein, with p.A275T and p.307_314del a part of putative caveolin-binding-like motif and leucine-rich repeats, respectively[22], 2 novel mutations(p.P.294L, p.G321Afs39X) were located in the joints between two transmembrane domains with p.P.294L and p.G321Afs39X a part of putative caveolin-binding-like motif and dimerization domain, respectively. These mutations might result in abnormalities of the secondary structure, which may change the protein functions. c.598-2A>C was also identified in another Chinese patients[15], but no further researches to testify its functions. Splicing site mutation has various consequences to RNA procession, including exon skipping, cryptic splicing, intron inclusion, leaky splicing, or introduction of pseudo-exons into the processed mRNA [23]. Results in our study revealed that the acceptor splicing site of *MLC1* was changed and the transcription of exon8 was inactivated, which might lead to a deletion of 39 amino acids from protein MLC1, impacting the structure and function severely. Compound heterozygous mutations were identified in Pt2 with a reported missense point mutation (p.S93L) and a splicing mutation (c.598-2A>C). We speculated the mutation (c.598-2A>C) was pathological, and Pt2 was genetically diagnosed. Deletion mutation from exon4 to exon9 found in Pt13 using MLPA test was larger than the previous reported heterozygous deletion of exon4 and exon5 in *MLC1*[2]. It might result in a large deletion of amino acids and might be pathological for Pt13. These results suggested *MLC1* dosage analysis was important when negative outcome occurred from *MLC1* and *GlialCAM* sequencing.

Homozygous mutations (c.772-1G>A, p.G73E), with heterozygous variation in mothers and wild type in fathers, were showed in Pt14 and Pt15, respectively. To explain those discrepancies phenomenon, firstly we excluded CNV (copy number variation) by *MLC1* dosage analysis using MLPA method (Data not shown); secondly maternal UPD were tested by haplotype analysis including 9 STR on chromosome22 and 8 STR on non-chromosome22 (Fig 5). Maternal and paternal alleles segregation of both non-chromosome22 and chromosome22 STR were found in Pt14 and indicated her homozygous mutation(c.772-1G>C) might be a *de novo* mutation or her father was mosaic. Otherwise, the result of Pt15 revealed maternal origin for two chromosome22 and maternal UPD was identified (Fig 5). UPD is the inheritance of both homologues of a pair of chromosomes from a parent, leading to trisomic mosaicism, genomic imprinting disorders, homozygosity for a recessive mutation or a combination of both latter conditions [24]. Homozygous mutation due to paternal UPD of chromosome22 had been reported in a metachromatic leukoencephalopathy (MLD) patient. In this study, we identified the first patient with MLC caused by maternal UPD of chromosome 22[25]. There was 25% risk of recurrence in the MLC families with regular autosomal recessive inheritance. But for Pt15’s family, it was much lower since UPD was a rare event with a frequency in newborns of 0.029% [24]. *MLC1* has founder effects in some ethnics [26], in our research, we found the mutation of c.772-1G>C in *MLC1* in 3 unrelated patients, together with 4 patients in other Chinese [7, 14], accounting for 15.5% (9/58) alleles, and it wasn’t found in other ethnics. However, c.772-1G>C in Pt14 was suspected to be a *de novo* mutation. c.772-1G>C might be a founder mutation in Chinese or a hot-spot mutation.

4(20%) were identified with mutations in *GlialCAM* with 3 mutations (p.K68M, p.R92W, p.T132N). Heterozygous p.A32V in *MLC1* and heterozygous p.R92W in *GlialCAM* were detected in Pt16 and both were inherited from his mother. Since *MLC1* is autosomal recessive inheritance mode, no MLC patients caused by a heterozygous mutation was reported, and p.R92W in *GlialCAM* was reported to be pathological [8], Pt16 was caused by heterozygous p.R92W in *GlialCAM*. We speculated him clinical improved and cranial MRI recovered. Unfortunately, we failed to obtain subsequent information of Pt16 and his families. Heterozygous p.R92W in *GlialCAM* was found in Pt17, his mother and maternal grandfather. Macrocephaly, motor development and classic cranial MRI were showed in Pt17, but only macrocephaly was described in his mother. Neither macrocephaly nor development delay were shown at his maternal grandfather’s young age by his families’ medical histories recalling. Different degrees of severity revealed in Pt17’s family might be a result of heterogeneity of MLC or unreliable memories of Pt17’s mother and grandfather [27–28]. 2 mutations (p.T132N, p.K68M) of *GlialCAM* occurred in the Ig-domain V-set where recessive mutations were identified previously in Pt19[8], changing the chemical properties of residues from hydroxyl (T) to amidic(N) for mutation p.T132N and from basic(K) to sulfureted(M) for the mutation p.K68M, which might alter the secondary structure and function of the protein, considered to be pathological.

Two heterozygous variations both inherited from his father while wild type in his mother in *MLC1* (p.S69W; p.I286M) was found in 1(Pt20) patient after sequencing the exons and the exon-intron boundaries of *MLC1* and *GlialCAM*, and the dosage analysis of *MLC1*. We failed to make genetic diagnosis for Pt20. His disease-causing mutations might result from deep-intron mutation or other genes related to MLC [29–30].

Based on the clinical and genetic characteristics, patients were divided into three types: type MLC1 was caused by mutations in *MLC1* with classic phenotype; type MLC2A was caused by homozygous/compound heterozygous mutation in *GlialCAM* with classic phenotype; type MLC2B was caused by heterozygous mutations in *GlialCAM* with atypical improving phenotype. According to this classification criteria, out of 20, 15(Pt1-15)(75%,) were type MLC1, 3(Pt16-18)(15%) type MLC2B, 1(Pt19)(5%) was type MLC2A, and 1(Pt19)(5%) might be type MLC1. This was the third time worldwide to report MLC patients with mutations in *GlialCAM* [8–9], and also the first time to identify mutations in *GlialCAM* in Chinese. Clinical severity varies among Pt24 and Pt25 siblings, Pt17’s families which were often seen in patients with the same mutation or even siblings [27–28], but similar features were detected in Pt9 and Pt10, Pt27 and Pt28 in this research.

## Conclusions

This clinical and genetic analysis for the patients with MLC has the largest samples in Chinese and it is also the first time to make follow-up study with the largest MLC patients. 20 patients were clinically diagnosed, with 17 patients classic phenotype, 2 patients atypical improving phenotype and 1(Pt16 missed) unclassified. Head circumference was ranged from 56cm to 61cm in patients older than 5yeas old, with a median of 57cm. Motor deterioration was found in 5 (18.5%) patients ranged from 2.5 to 11 years old. Cognitive deterioration occurred in 2 patients (7.4%). Motor ability improvements and cranial MRI recovery were first found in Chinese patients. Rate of seizures (45.5%), transient motor retrogress (45.5%) and unconsciousness (13.6%) after minor head trauma was much higher than that after fever (18.2%, 9.1%, 0%, respectively). 10 novel mutations in *MLC1* were identified, expanding the spectrum of mutations. *GlialCAM* mutations were identified in 4 patients. Splicing mutation of c.598-2A>C in *MLC1* was firstly confirmed to lead to the skip of exon8. MLPA was firstly applied in *MLC1* dosage analysis and homozygous deletion mutation from exon4 to exon9 was identified firstly in MLC patient. Homozygous point mutation due to UPD was firstly discovered in MLC patient. c.772-1G>C in *MLC1* might be a founder mutation or a hot-spot mutation was likely to be a founder mutation in Chinese or a hot-spot mutation.
